# Supplementary material for: Are carbon emissions trading and green financial instruments synergistic? -Comprehensive quantitative research based on content analysis
Source: PLoS One. 2024 Mar 7;19(3):e0298601. doi: 10.1371/journal.pone.0298601 (PMC10919601; doi:10.1371/journal.pone.0298601)
Supplement: S1 Data — (ZIP) [file pone.0298601.s001.zip › Data/地级市Z_F工作报告.zip!/║╙▒▒/└╚╖╗╩╨/└┤╫╘╣1⁄2╓┌║┼í╛┬φ┐╦╩2╛▌═°í┐_2015.docx]

各位代表：

现在，我代表市人民政府向大会报告工作，请予审议，并请市政协委员和列席会议的同志提出意见。

一、2014年工作回顾

刚刚过去的2014年，是廊坊发展史上很不寻常的一年。面对严峻复杂的形势和艰巨繁重的任务，在省委、省政府和市委坚强领导下，我们准确把握京津冀协同发展大势，深入开展“十项集中行动”，统筹抓好稳增长、调结构、促改革、优生态、惠民生各项工作，较好地完成了市六届人大二次会议确定的目标任务。

1.综合实力显著提升。主要指标实现进位，增速均居全省前列。全市生产总值2056亿元，同比增长8.2%，增速居全省第1位，比上年提升2位；全部财政收入406.7亿元，增长16.5%，总量和增速分居全省第4位和第1位；公共财政预算收入250.5亿元，增长21.9%，总量和增速分居全省第3位和第1位；全社会固定资产投资1882.2亿元，增长19.3%，增速居全省第1位，提升2位；规模以上工业增加值705.5亿元，增长8.1%，增速居全省第3位，提升4位；社会消费品零售总额716.1亿元，增长12.3%；实际利用外资7.17亿美元，超额完成任务。经济结构更趋优化，质量效益稳步提高。全部财政收入占生产总值比重达到19.8%，税收占全部财政收入比重达到92%，均居全省第1位；规模以上高新技术产业增加值占规上工业增加值比重达到19.1%；服务业对经济增长的贡献率首次突破50%。

2.产业结构持续优化。坚持抓投资上项目，以增量优化带动存量调整，产业结构出现积极变化。项目质量明显提高。全市建设亿元以上项目913项，同比增加56项，其中10亿元以上项目170项，增加37项，新兴产业项目占比达到40%，项目投资强度、税收贡献都有了明显提升。争列省重点项目13项，居全省第1位。润泽信息港、精雕数控机床一期、泉恩管材等一批大项目建成投产；机器人产业港、中集空港设备、卫星导航产业港等一批战略性新兴产业项目基本建成；碳纤维新材料、微软游戏创新中心等一批高端项目加快建设。新兴产业快速发展。电子信息产业持续壮大，华为、富智康等既有企业增势不减，联通华北基地、鼎威液晶显示等一批新项目加快建设，全年实现营业收入278亿元，同比增长10%。先进制造业加快发展，维特根、航天振邦、日上门业、立马电动车等重点项目实现扩大再生产，航天技术应用产业基地、镁合金高铁轻量化部件等一批新项目加快建设。新增规上工业企业160家。现代服务业增势强劲，鲁能生态区、大城红木家具城等项目带动作用持续增强，港中旅海泉湾、胜记仓、新朝阳二期、京东商城华北订单处理中心等一批新项目加快建设，服务业增加值增长12.2%，居全省第1位。现代农业蓬勃发展，达利食品、汇福粮油、太古冷链、新苑阳光等现代农业项目势头强劲，新增农业高效节水面积15万亩，农业产业化经营率提高到67%。传统产业升级步伐加快。大力实施“四个五十”工程，市县两级设立技改专项资金3亿元，以政银企合作新模式撬动金融资本，实施技改项目604项，完成工业技改投资573亿元，增长29%，居全省第1位。园区平台更趋高端。加大园区整合提升力度，新增4家省级高新区，省级以上高新区数量居全省第1位。新增1家省级经济开发区和1家省级文化产业示范区。省级以上园区财政收入、利用外资、固定资产投资分别占到全市的72%、87%、75%。

3.生态环境稳步改善。坚持生态优先发展理念，突出重点，综合施策，坚决向污染宣战。大气污染治理成效明显。组织开展“八大攻坚战”、“蓝天行动”和淘汰产能拆除集中行动，全市淘汰767台工业燃煤锅炉和236台燃煤茶浴炉，削减燃煤54.8万吨，淘汰黄标车6.6万辆，加油站全部安装油气回收装置。超额完成省下达淘汰过剩产能任务，关停拆除相关企业178家，淘汰水泥产能1603万吨、造纸1.5万吨、玻璃330万重量箱、岩棉21.1万吨。炼铁、炼钢产能分别压减115万吨和72万吨。圆满完成APEC空气质量保障任务。植树增绿力度空前。全年造林57.35万亩，一年超额完成四年省定任务，市区新增绿地113万平方米。生态修复全面实施。编制完成廊南森林湿地生态走廊建设规划，永定河、潮白河等河流综合治理加快推进，胜芳和文安湿地公园建设全面启动，香河湿地公园列入国家级湿地公园试点。节能减排任务全面完成。PM10、PM2.5、二氧化硫、一氧化碳浓度同比下降13.6%、9.1%、21.7%和18.2%。全年空气达标天数153天，同比增加21天，重污染天气减少14天。

4.协同发展实质推进。制定出台加快推进京津冀协同发展思路框架和行动方案，主动对接，积极融入，各项工作取得新进展。重大利益诉求得到体现。紧盯国家顶层设计动态，加强重大问题研究，与国家部委、京津部门和高层智库充分沟通协调，廊坊城市、产业功能定位等重大利益诉求得到体现。重点领域率先突破。功能疏解方面，北京八中固安分校建成使用，燕达医院与北京朝阳医院实行共建共管。产业承接方面，中兴研究院建成投用，中海油产业化基地开工建设，中核研发中心成功签约，华融金融后台服务中心、社科院考古基地等一批高端转移项目加快推进。交通对接方面，京廊交通规划一体化和北京新机场交通集疏运体系建设达成共识，京廊通勤高铁“廊坊号”开通运行，京台高速廊坊段建成通车、北京段启动拆迁，密涿高速、津保高铁建设进展顺利，京秦高速及国省干道对接加快实施，廊涿城际、京九客专、津石高速规划设计工作全面展开。与通州就建设“四路一河”达成共识，潮白河大桥对接工程完成前期工作。合作模式实现创新。与北京经济技术开发区合作共建亦庄·永清产业园，双方共建筹委会办公室已全面启动，首批6个项目即将开工建设。与北京西城区共建现代物流园区选址和规划编制已基本完成，永清国际服装城建设进展顺利。清华大学重大科技项目中试孵化基地开工建设，北京大学非人灵长类动物试验中心建成投用，香河与清华大学共建了研发教学实践基地。两大“引爆点”加快推进。北三县统筹发展实质推进，统筹发展规划和四个专项规划编制完成，域内交通互联、产业布局、生态共建迈出新步伐。北京新机场开工建设，廊坊空港投资开发公司完成组建。

5.改革创新步伐加快。坚持把改革创新作为发展的根本动力，着力破除体制机制障碍，发展空间更为广阔。重点领域改革取得新成效。大力推进简政放权，衔接落实省取消下放行政审批事项27项，市县政府机构改革顺利推进，全面完成食药监管体制改革，在全省率先实现市直部门机关标准化管理，“一办两中心”规范运行，负面清单制定发布，“三级平台”全面建成，“两个代办”高效实施，办事效率和服务质量明显提升。积极稳妥推进“营改增”试点扩围，为企业减税6.45亿元。商事制度改革持续推进，113个前置审批事项改为“先照后证”，新增市场主体3.2万户，同比增长49.4%。农村集体土地确权登记、农村土地承包经营权确权登记颁证扎实推进，完成农村土地承包经营权流转面积71万亩。科技、教育、文化等社会领域改革取得新进展。金融活力显著提升。全市存贷款增量、增速均居全省前列。中国华融、中信国安及9家省级银行机构与我市签署战略合作协议，新增4家村镇银行，市小额票据贴现中心挂牌运营。全市新增挂牌上市企业20家，宏泰发展在香港联交所主板成功上市。强力管控金融风险，果断处置一批非法集资问题。对外开放迈出新步伐。开展招商引资集中行动，签约亿元以上项目623个。编制完成7个重点产业招商路线图，择商选资的针对性、实效性进一步提升。成功举办“5·18”经洽会、“9·26”农交会。创新能力持续增强。深入实施科技创新“十大工程”，新增科技型中小企业518家，新增规上高新技术领域企业15家，累计达到266家。新增企业研发中心17家、院士工作站2家。廊坊科技企业创新创业园基本建成，全市科技孵化器达到50万平方米。投入创投资金4420万元，撬动金融信贷和社会资金2.5亿元，有力支持了科技型中小企业发展。专利申请量突破2000件，专利授权1691件。顺利通过国家可持续发展实验区验收。民营经济快速发展。全市民营企业实缴税金占全部财政收入比重达到89%，新增注册企业2350家。我市连续四年被评为“河北省民营经济发展先进市”，四个县（市）跻身全省民营经济十强县。

6.城乡统筹扎实推进。坚持以规划为龙头，城乡一体、统筹推进，加快新型城镇化进程。城镇功能更趋完善。《廊坊市城市总体规划》编制完成。中心城市承载力全面增强，投资80亿元实施城建十大工程，自然公园西区正式开园，生态文化艺术新区初具规模，“梦廊坊”文化产业园加快建设，主城区70条小街巷实现提升改造，古县路、新开路续建工程竣工通车，完成14个无物业旧小区改造，城市精细化管理水平持续提高。县城建设加快推进，县城规划编制全部完成，投入25.4亿元开展城市道路、配套设施建设，县城建设水平大幅提升。全市城镇化率达到53%以上。城乡基础设施加快建设。103、104国道等重点改造工程全部完工。南水北调天津干渠实现通水，廊涿干渠全面试水，保沧干渠主体完工，8座地表水厂加快建设，北三县调水工程获省批准。争取引黄指标1亿立方米，对廊南地区生态修复和湿地建设意义重大。完成110千伏及以上输变电工程4项，市城区实现220千伏双环网供电。农村面貌改造提升行动扎实开展。30个市级精品村和428个省级重点村的改造提升任务全面完成，积极利用新技术、新材料、新装备、新样式改善农村人居环境，各项工作走在全省前列，省委、省政府召开会议推广我市做法。

7.人民生活持续改善。加快以改善民生为重点的社会建设，把更多公共资源用于民生保障薄弱环节，民生支出占公共财政预算支出的80.9%，比上年提高3个百分点。民生保障力度持续加大。城镇、农村居民人均可支配收入分别达到29416元和12115元，分居全省第1位和第2位。城镇登记失业率2%，连续9年全省最低。培训农民22.7万人次，实现农村劳动力转移就业2.98万人。城乡居民养老保险参保率98.9%，新农合参合率97.6%。城镇、农村人均低保标准分别增长26%和25%，保障标准居全省首位。启动75所中小学、幼儿园建设，其中25所投入使用。保障性安居工程开工1.71万套、竣工1.07万套，超额完成省定任务。

市区新增6条公交线路，新购置93辆新能源公交车投入运营。投资1.3亿元，解决28.7万农村居民饮水安全问题。社会事业加快发展。在全国率先实现新生儿35种遗传病免费筛查全覆盖，在全省率先编制区域卫生规划。全面完成县级公立医院改革，7个县（市）被确定为国家级试点，政府办基层医疗卫生机构全部配备基本药物。“单独两孩”政策启动实施。国家公共文化服务体系示范区创建工作加快推进。市区建成22公里绿道健身工程，农村体育健身设施覆盖率达到53.8%，在省运会、残运会、少数民族运动会上取得好成绩。高度重视残疾人就业、培训、救助等工作，荣获全省残疾人技能大赛团体第一名。大厂回族自治县被国务院授予“全国民族团结进步模范集体”荣誉称号。和谐稳定局面得到巩固。全面落实新《安全生产法》，事故起数和死亡人数双下降，安全生产形势平稳。信访积案化解集中行动深入开展，平安社区创建成为全省试点市，圆满完成十八届四中全会等重大活动安保任务。国防动员、妇女儿童、防灾减灾、外事侨务、新闻广电出版、气象、地震、人防、援疆、档案等各项事业取得新成绩。

一年来，按照市委的统一部署，在全市政府系统深入开展党的群众路线教育实践活动，持续聚焦“四风”突出问题，严格落实中央“八项规定”，作风建设形成新常态。启动实施了“十二件惠民实事”，扎实开展了“跟踪回访、问效于民”和“五诺一满意”活动，解决了一批群众反映强烈的突出问题。积极推进廉洁政府建设，严格落实党风廉政建设主体责任，持续强化行政监察和审计监督，依法行政能力不断增强，政府自身建设迈出新步伐。自觉接受市人大、市政协监督，办理人大代表建议和政协委员提案494件，办复率均为100%。开通961890群众服务热线，实现24小时接听，及时解决了一大批群众反映的困难和问题。

各位代表！过去的一年，我们经历的大事多、新事多、难事多，面临的期望高、责任大、任务重，特别是化解过剩产能、治理大气污染、APEC空气质量保障等方面带来的压力集中交织、困难前所未有。面对风险挑战，我们坚持“绿色、高端、率先、和谐”发展定位，积极适应经济发展新常态，努力转变推进发展的思维理念、方式方法，更加注重提升发展质量和效益，更加注重改革开放和创新发展，更加注重工业强市和服务业兴市，更加注重环境治理和生态修复，全力为廊坊长远发展打基础、强支撑、增活力，经济社会保持了良好发展态势。成绩来之不易，这是省委、省政府和市委科学决策、坚强领导的结果，是市人大、市政协有效监督、大力支持的结果，是全市上下同心同德、拼搏进取的结果。在此，我代表市人民政府，向全市人民，向人大代表、政协委员，向各民主党派、工商联、无党派人士和人民团体，向驻廊解放军、武警官兵、政法干警和中省直驻廊单位，向所有关心支持廊坊发展的朋友们，致以衷心的感谢和崇高的敬意！

我们也清醒地看到，我市经济社会发展中还存在不少矛盾和问题。经济下行压力加大，一些企业生产经营困难。转型阵痛加剧，传统产业占比高，新兴产业支撑弱，创新能力不足。生态环境约束趋紧，雾霾治理和生态建设任务艰巨。资源要素制约突出，融资难、用地难加剧，金融风险加大。发展环境有待提升，园区和城市承载能力不足，公共服务能力不强。社会民生欠账较多，群众关心的热点难点问题还没有得到很好解决。一些政府部门和工作人员依法行政和服务意识淡薄，一些领域和地方消极腐败现象还比较严重。面对这些矛盾和问题，我们一定直面应对、勇于担当，以更大的决心、更大的力度、更有效的措施认真加以解决，决不辜负全市人民的期望！

二、2015年工作总体要求

今年是全面完成“十二五”规划的收官之年，是全面深化改革的关键之年，也是全面推进法治廊坊建设的开局之年，做好今年的工作意义重大。当前，国内外经济形势依然严峻复杂，不确定因素依然较多，经济下行压力依然较大。我市处于转型发展的关键时期，面临区域竞争更趋激烈、转型升级任重道远、财政收支矛盾加剧等多重困难和挑战。同时也要看到，我市发展迎来一系列重大机遇，我国已进入经济发展新常态，一系列政策措施的出台，有利于我们顺势而为、加速发展。京津冀协同发展全面推进，北京新机场、临空经济区等重大项目加快实施，重点领域协同加速突破，我们原来想都不敢想的事、想做做不成的事正一件件成为现实，廊坊真正到了全面突破、跨越赶超的关键时期。面对新形势新任务新要求，我们必须以更加强烈的责任感和使命感，保持定力、主动作为，攻坚克难、奋发有为，努力开创廊坊发展新局面。

今年政府工作的总体要求是：全面贯彻党的十八大、十八届三中四中全会和习近平总书记系列重要讲话精神，认真落实中央、省经济工作会议和市委重大部署，紧紧围绕“绿色、高端、率先、和谐”发展定位，牢牢把握京津冀协同发展重大机遇，坚持稳中求进工作总基调，以提高经济发展质量效益为中心，主动适应经济发展新常态，把转方式调结构放到更加重要位置，着力强化改革攻坚，更加突出创新驱动，全面优化生态环境，切实加强民生保障，扎实推进依法行政，促进经济持续健康发展和社会和谐稳定。

今年经济社会发展主要预期目标是：全市生产总值增长8%以上，公共财政预算收入增长11%，规模以上工业增加值增长8%，固定资产投资增长19%，社会消费品零售总额增长12%，服务业增加值增长12%，城镇、农村居民人均可支配收入分别增长8.5%和9%，实际利用外资增长5%。单位GDP能耗、主要污染物减排和空气质量改善绩效指标完成省下达目标任务。这一指标安排，主要是与“十二五”规划和全面建成小康社会目标相衔接，符合我市实际和社会预期，旨在着力提高经济发展质量效益、调整优化经济结构，促进经济社会持续健康发展。

做好今年的政府工作，必须把转变发展理念与强化工作落实结合起来。深刻认识经济发展新常态的特点和规律，坚定不移走改革之路、创新之路、法治之路，把新理念、新思路转化为规划、项目和实际举措，掷地有声抓好落实。必须把加快发展与提高质量效益结合起来。紧紧抓住千载难逢重大机遇，在优化结构、强化质量效益的基础上，努力实现与廊坊区位、条件相匹配的快速发展、跨越发展。必须把大力发展新兴产业与改造提升传统产业结合起来。坚持项目带动、创新引领、集群集约，培育壮大战略性新兴产业，加快传统产业转型升级，努力打造廊坊产业升级版。必须把经济发展与保障民生结合起来。坚持把更多公共资源向民生薄弱环节倾斜，更加注重提升公共服务、增进民生福祉，全力维护社会公平正义。只要我们定下心、铆足劲，凝心干事业，聚力抓发展，就一定能完成好今年各项任务。

三、2015年主要工作任务

今年我市改革发展稳定的任务艰巨而繁重，必须狠抓关键，全力攻坚，着力做好九个方面的工作：

（一）大力推进协同发展。在更广领域、更高层次深化与京津对接合作，在京津冀协同发展中继续走在全省前列，努力建设京津冀协同发展先行区。

加快制定实施方案。积极配合国家和省编制京津冀协同发展总体规划和专项规划，进一步明确廊坊作为首都城市功能拓展区、京津产业转移提升承载地、全国科技创新成果孵化转化示范区、“京津走廊”生态宜居城市的定位。启动编制我市协同发展实施方案，落实好规划确定的重大事项，争取国家和省在我市开展区域协同发展的试点示范。

推进交通互联互通。力促廊涿城际、京九客专等轨道交通项目开工建设，确保津保高铁廊坊段建成通车。加快密涿高速建设进度，推动津石、唐廊等高速项目尽快实施。加大协调力度，以推动京台高速北京段加快建设、102国道南移、京秦高速与京津对接、跨潮白河大桥建设等为重点，尽快打通与京津“断头路”，力争京津廊三地交通互联实现新突破。

强化产业对接。加快亦庄·永清产业园建设，创新园区建管体制和运作模式，高标准编制园区规划，全力建设园区基础设施，力促首批入园项目尽快开工，努力建成京冀产业协同示范区。加快中关村固安大清河开发区和永清西城物流园、国际服装城等一批合作园区建设，加快已开工项目建设进度，力促已签约项目尽快落地，力争再引进一批重大产业项目。以更大力度做好首都教育、医疗、会展等功能性资源引进承接工作。

（二）积极构筑区域发展新格局。着眼实现资源要素科学布局、高效配置，进一步创新发展思路、完善推进机制，统筹推进北、中、南三大区域协调发展。

以区域一体化为目标，加快北三县统筹发展。突出规划引领。全面执行《北三县统筹发展规划》，推进控制性规划和专项规划全覆盖，高标准编制沿潮白河高新技术产业带总体规划、核心起步区规划，启动北三县东部区域规划编制。坚守生态红线，控制人口规模，划定城市边界，促进交通、生态、产业、城镇协调发展。做大做强燕郊高新区。提升创新能力，加快科技成果孵化转化核心基地建设，力促燕郊高新区向国家一流高新区迈进。推动燕郊高新区扩区，改革管理体制，带动提升三县市现有园区平台，共享高新区优惠政策，打造沿潮白河高新技术产业带。增强核心区支撑作用。将沿潮白河高新技术产业带大厂园作为北三县统筹发展核心区，引入战略投资者，重点推动中央商务区、生态景观和功能配套区建设，力争一年出形象、两年成规模，为北三县产业发展提供支撑平台。

以北京新机场和临空经济区为依托，打造中部战略增长极。全力推进北京新机场和临空经济区建设，重点发展现代服务业和高新技术产业，发挥对中部区域的引领作用。做好机场征迁安置和施工保障。坚决完成各项征迁安置任务，加快推进天堂河改道、白家务水源地迁建、永定河滞洪区调整等配套项目，积极做好供油、空管、军事阵地迁建和高压线迁改等各项工程的配合工作。加强新机场综合交通体系规划建设。加快编制廊坊至机场轨道交通路由及站场设置方案，力促廊坊至机场高速公路项目与北京市签订接线协议，制定市域干线公路与机场衔接方案。加快临空经济区建设。密切跟踪国家新机场临空经济区规划进展，加强与国家和省有关部门沟通联系，争取更多体现廊坊利益诉求。做好我市临空经济区总体规划、起步区规划、土地利用和产业布局规划编制工作，适时启动起步区道路及相关市政基础设施建设。

以建设现代制造业基地为重点，力促南三县加快崛起。充分发挥南三县民营经济发达、制造业基础较好的优势，以强园区、上项目、畅交通、优生态为重点，集中力量打造现代制造业基地。坚持创新驱动，做好“有中生新”、“无中生有”两篇文章，着力解决传统产业层次低、新兴产业实力弱的突出问题，做大做强木材加工、家具制造、保温建材、食品加工等传统产业集群，推动电动车、气雾剂、高铁轻量化部件、易拉罐等新兴产业集群式发展，下大功夫引进培育一批先进制造业和战略性新兴产业项目。认真谋划实施一批对接京津、连通域内的交通路网项目，持续改善生态环境，为南三县加快发展提供基础支撑。

（三）深入实施创新驱动战略。围绕建设科技之城、创新之城目标，以产业创新为重点，以协同创新为路径，深入实施“一十百千万”科技创新工程，打造全国科技创新成果孵化转化示范区。

加快编制全国科技创新成果孵化转化示范区规划。着眼全域规划、统筹推进，以国家级、省级高新区和现有园区为节点，打造以战略性新兴产业、现代服务业为特色的环首都科技创新带，以特色产业提升、高端装备制造相结合的环天津科技产业带，走出一条京津研发、廊坊孵化中试到生产制造的发展之路。

打造十大创新平台。以提升廊坊对优质科技资源的吸附力、承载力为目标，集中力量建设园区承载、创业孵化、成果转化、科技金融、产学研合作、企业研发、创新服务、产权交易、科技招商、政策支撑十大平台。每个园区至少建设一个1万平方米的科技孵化器，全市达到100万平方米。推进廊坊科技型中小企业创新创业孵化园、肽谷生物医药孵化园、龙河科技成果孵化园等10家共建重大科技成果转化基地建设，新建3家院士工作站、2家博士后工作站，新增企业研发机构100家。用好科技金融政策，搞活创投基金，持续加强财政金融支持。建设科技成果交易、展示、服务中心。

建设百个产业集群。依托京津科技资源，大力推动协同创新，以产业链布局创新链，以科技龙头聚集创新项目，加快培育引进一批科技龙头企业，推动以润泽信息港为龙头的大数据、新奥集团为龙头的清洁能源、卫星导航产业港为龙头的航天技术应用、微软游戏创新中心为龙头的电游产业、机器人产业港为龙头的机器人制造等创新型产业集聚发展，打造100个产业集群。

培育千家科技型中小企业。通过孵化器培育一批、科技人员创办一批、科技招商引进一批，做大群体，做强实力，做优结构，年内新认定科技型中小企业1300家，科技小巨人企业达到150家。

引进万名科技创新人才。实施高端人才引进计划、科技人员创业计划、科技人才培养计划，推行科技成果产权制度改革、股权和分红激励等政策，培育引进一批支撑产业创新发展的优秀创新群体。全年引进10000名科技创新人才，专利申请量突破3000件。

围绕实施“一十百千万”工程，制定实施方案，召开全市创新发展大会，成立市科技创新工作领导小组，将科技创新主要指标纳入县（市区）和园区考核体系，形成推动科技创新的强大合力。

（四）坚持不懈优化生态环境。把生态红线作为发展的生命线，以更大决心、下更大力气推进环境治理，努力建设绿美廊坊。

打好大气污染治理攻坚战。强力开展大气污染治理集中行动，实施“控煤、抑尘、限车、治理工业污染、治理各类烟气”五大工程。以治理燃煤污染和面源污染为重点，大力推广微煤雾化等煤炭清洁燃烧技术，市城区基本淘汰10蒸吨及以下燃煤锅炉、茶浴炉。对没有纳入集中供暖的中小学、机关事业单位及无物业分散供暖小区实施治理改造，对15万户城中村居民和农村居民开展清洁炉具改造替代。推进钢铁、水泥、电力、玻璃四大行业国家重点治理工程，全部淘汰黄标车，建立农村秸秆禁烧长效机制。加快国电热电联产项目建设。

持续提升全域绿化水平。以创建全国平原森林城市为目标，进一步创新机制、强化举措，提升造林标准和景观效果，持续推进生态廊道、规模化造林、村庄绿化、环京津边界绿化、重要交节点绿化、城镇绿化、名优林果苗木基地、高标准农田林网八项重点工程。全力打造精品廊道，实现廊涿、廊沧、京台等县域之间重点线路的绿化闭合。全年造林50万亩以上，力争森林覆盖率提高5个百分点。

加快推进生态修复工程。全面加强湿地建设，以“一轴五河六点”为脉络，打造廊南森林湿地生态走廊，恢复湿地7万亩。沿永定河左堤建设郊野公园等节点景观。开展重点河流治理攻坚行动，加快潮白河、北运河、龙河等河流治理工作。南水北调地表水厂上半年全部建成投用，北三县调水项目完成可研和初步设计，尽早开工建设。实现引黄调水常态化。落实最严格的水资源管理制度，加强地下水位监测网、取用水监测网、地表水监测网、水质监测网和水资源监管平台建设，实现全域水资源动态监控。加快铁路北污水处理厂建设，启动万庄新城再生水厂工程，完善已建成污水处理厂配套管网。加强三河东部矿山环境治理。

完善污染防控体制机制。全面落实新《环境保护法》，依法实施源头严控、过程严管、后果严惩，推进防治工作长效化、法治化。深化与京津大气联防联控，推动生态共建共享。完善基层环保工作网格化监管和智慧环保建设，建立环保、公安双重管理的执法体制，严厉打击环境违法行为。

（五）全力上项目增投资。坚持把项目作为增实力、优结构的核心抓手，以迎接省重点项目拉练观摩活动为契机，全力推进项目建设取得新突破。

加大项目建设力度。争列省级重点项目继续保持全省前列，建设市级重点项目100项，实施亿元以上项目1000项以上。建立健全项目建设推进机制，以投资强度、科技含量、税收贡献率和建设容积率为衡量标准，确保项目质量。加快项目建设进度，开展两次项目拉练观摩活动，建立项目台账，逐一督导落实，重点项目开工率、投资完成率走在全省前列。

破解项目要素制约。把市级统筹和发挥各级各部门、项目业主积极性结合起来，多途径破解土地制约，大力度清理闲置土地，制定鼓励企业集约节约用地政策措施，优先保障好项目、大项目土地供应。加快特高压电力工程建设，确保完成30项110千伏以上输变电工程，确保电力保障。加大项目调度力度，帮助解决项目融资问题，确保资金供应。

提高项目招商实效。开展招商引资集中行动，按照产业招商路线图，实施以商招商、园区招商、定向招商、委托招商、专业招商，全面强化招商针对性、精准性和实效性，力推产业“建链、补链、强链”，打造产业集群。办好“5·18”经洽会、“9·26”农交会等招商活动。

提升项目承载平台。发挥国家级园区带动作用，加大廊坊开发区和燕郊高新区改革创新力度，推动政策外溢共享，带动其他园区发展。加快园区整合提升步伐，新建一批省级高新区，支持京南·固安高新区申报国家级高新区。加强国别园区建设，力争新建2家以上国别园区。着力完善园区科技成果孵化、创新创业基金等服务体系，集中整合优势资源，打造一批功能完备、特色鲜明、承载力强的品牌园区。

（六）加快推进产业转型升级。按照一产要优、二产要强、三产要大的思路，积极构建绿色循环低碳现代产业体系，打造新兴产业集聚区。

加快工业强市步伐。推动产业集群发展。大力培育引进行业龙头，规划建设专业园区，以龙头带动、链条延伸、园区聚集为路径，推动产业集群集约发展。筛选航天技术应用、智能机器人等发展前景好、综合效益高的战略性新兴产业，进行重点培育、重点支持，迅速壮大产业规模，努力实现爆发式增长。拓宽企业融资渠道。积极引导企业上市融资，瞄准多层次资本市场，支持优质企业挂牌上市。积极争取国家、省产业专项资金、基金，扩大市级产业扶持资金规模，加强政银企合作，采取委托贷款、股权投资等形式，支持产业升级发展。加强技术引进对接。依托省内外高校院所和重要展会，搭建技术引进对接平台，支持企业建立技术研发机构，提升企业技术水平。全市新增规上工业企业150家以上。

实施服务业兴市战略。优先发展生产性服务业。全力推进清华大学科技成果中试孵化基地、中兴研究院、京东商城华北订单处理中心、366网上商城等重点项目，加快建设人保北方信息中心，积极打造金融后台服务中心，着力培育壮大研发设计、现代物流、电子商务、金融服务、商务会展等与工业转型升级密切相关的生产性服务业。积极发展生活性服务业。围绕文化旅游、健康养老、社区服务、家政服务等消费新热点，推动生活性服务业加速成长。建立全市股权多元化文化产业引导基金，以“梦廊坊”文化产业园、国华影视基地等为依托，打造文娱体验、展演、创作、拍摄为一体的文化体验高地。积极开展旅游产业推介招商，面向京津市场，打造旅游品牌，力促旅游人数突破2000万人次，增长20%。以中国（固安）单车运动中心等项目为龙头，推动健康休闲业快速发展。以燕达健康城等项目为依托，发展医养结合养老基地。加大限额以上企业培育力度。像抓规上工业企业一样抓好限上企业，全年新增限上企业60家，入统企业达到400家。

大力发展现代农业。围绕都市型农业发展定位，像抓工业园区一样抓现代农业园区，加快推进固安、大厂等省级现代农业园区建设。引导发展农民合作社、家庭农场等新型农业组织。着力建设一批国内先进的高效节水示范园区，发展高效节水农业15万亩。做大做优蔬菜、粮油、肉类等优势产业，加快食品工业发展。加快建设北方国际万果园、太阳能光伏农业基地等一批现代农业项目，引领现代农业高端化发展。

（七）全面推进深化改革。坚持问题导向，实施重点突破，通过深化改革破除各种障碍、增强发展活力。

稳妥推进重点领域改革。推进行政体制改革。做好国务院和省取消下放行政审批事项衔接，继续取消下放一批行政审批事项。完成市县政府机构改革，优化市县政府机构设置，推行县以下机关公务员职务与职级并行制度。继续深化商事登记制度改革，强化事中事后监管。深化国企改革。完善国企监管体制，以管资本为主全面落实国资监管责任。进一步完善企业法人治理结构，加强董事会建设，建立健全监事会外部监管体制。积极稳妥推进混合所有制经济发展。实施农村综合改革。推进农村产权制度改革，推动农村土地承包经营权流转和农民专业合作组织发展，力争农村土地承包经营权确权登记颁证完成50%以上。深化社会事业改革。稳步推进机关事业单位养老保险制度改革，加快供销社和县级公立医院综合改革，进一步提升就业、教育、社保等公共服务水平。加快社会信用体系建设。强力推动政务、商务、社会、司法等重点领域信用分类监管、信息共享和联动惩戒。

积极推进财税金融改革。严格落实新《预算法》，依法编制收入预算，改进控制机制，提高财政绩效。进一步扩大“营改增”范围。加强银企对接和金融创新，加大对中小微企业、涉农企业的融资支持。加强地方金融监管体制改革，开展各类投资、小贷、典当、担保等专项治理行动，坚决打击非法集资，防范和化解金融风险。

大力推进民营经济发展。健全中小企业服务体系，强化融资服务、项目推介、市场开拓、创业辅导等功能，继续实施民企服务集中行动，选取100家成长潜力大、科技含量高、产业带动强的民营企业，由市县两级领导分包服务。落实好鼓励全民创业政策，建立中小企业发展专项资金，推动园区建设中小企业创业园。全年新增注册民营企业2000家以上。

（八）积极推进新型城镇化。以建设宜居宜业明星卫星城为目标，以智慧城市建设为依托，坚持产业、生态、文化融合发展，加强城乡建筑格调色彩研究，努力建设新型城镇化和城乡统筹示范区。

推进智慧城市建设。制定完善智慧廊坊发展规划和建设计划，统筹推进信息化与工业化、城镇化融合发展。积极运用新一代信息技术，对政务、经济、社会、民生等各领域进行分类谋划，着力建设智慧城管、智慧交通、智慧社区、智慧民生等各类平台，提供高效便捷的智慧应用和综合服务。

做大做强中心城市。以提升城市品位、完善城市功能为目标，围绕新区开发、旧城提升、交通路网、市容环境、园林绿化等，实施城建十大工程。启动实施北凤道西延、金光道西延、外环路改造等7个路网项目，实施自然公园西扩、高铁站广场、城市北出口等10个绿化提升工程，加快城市水系建设，实施步行街整修改造。推进市区组团发展，力争廊坊城市总规尽快获批，适时启动永清、固安撤县设区工作。推进市区管理体制改革，积极稳妥下放城市管理权，构建“两级政府、三级管理、四级落实”管理体制。强化城市精细化管理，拓展数字城管监管范围，扩大城市管控系统和网格化管理覆盖面。

提升县城和重点镇承载力。加快编制县城控制性详规、产业规划、土地利用规划等专项规划，加快重点镇和重点村街的规划工作，提高全域规划水平。加快社区服务体系建设，完善社区功能，加强管理创新。加强县城道路、供水供热、垃圾处理、绿化亮化等基础设施建设，完善文化、娱乐、医疗、教育等功能设施，打造一批县城样板街道。加快永定河泛区安次安全区和临空安全区建设，推动永定河泛区永清安全区、文安洼左各庄安全区等早日立项，为发展拓展空间。

扎实推进新农村建设。深入开展农村面貌改造提升行动，巩固提升1105个已改造村街，突出抓好290个新列入的省级重点村街。加速构建环京津美丽乡村示范区，北三县、固安县按照全省一流标准，成方连片建设省级精品示范区，其他县（市区）打造市级精品示范片。实施新农村建设“十县百村工程”，以拆迁复垦为重点，全面推进中心村新民居建设，改善农村面貌。

（九）尽心竭力保障改善民生。按照保基本、兜底线、促公平的要求，全力加快以民生为重点的社会建设，重点实施九项提升工程，持续提高人民群众幸福指数。

就业提升工程。落实促进就业创业政策，完善城乡均等的公共就业服务体系，应届高校毕业生就业率达到87%以上，新增城镇就业5万人，城镇零就业家庭动态归零，城镇登记失业率控制在4%以内。培训农民20万人次，实现农村劳动力转移就业2.8万人。

社保提升工程。完善多层次社会保障体系，进一步扩大社会保险覆盖面，提高社会保险待遇水平。推进城镇基本医疗保险市级统筹、工伤保险省级统筹，推进城乡居民大病商业保险，建立疾病应急救助机制。新农合人均筹资标准达到490元，财政补助由320元提高到380元。加快建设全国养老服务综合改革试点城市，实现社区居家养老服务站全覆盖、城镇困难老人补贴全覆盖。以提供早餐、提高待遇、设立爱心驿站为重点，开展环卫工人关爱行动，举办环卫工人节。

医疗卫生提升工程。新建市中心医院完成主体工程。市区公立社区卫生服务中心完成2所新建、3所续建任务。全市建设国医堂15个，指导带动540个中医药示范村卫生室和6个社区卫生服务站建设。继续免费为全市新生儿提供遗传病筛查。

安居提升工程。开工建设保障性住房1.6万套。加快管道局旧小区等25个连片棚户区、老旧小区的改建工作。加大农村危房改造力度，强化农村住房安全。

教育提升工程。完成市一中迁建主体工程。市区完成5所中小学、3所幼儿园改扩续建。实施248所农村义务教育薄弱学校改造工程，创建标准化学校120所，50%以上中小学校实现标准化。全面提升农村幼儿园建设、管理和办学水平，新建一批农村幼儿园，坚决取缔无证幼儿园，力争用两年时间基本改善农村幼儿园办学条件。

便民设施提升工程。市区启动实施5条小街巷、1个市场路面改造和20个便道、32个路口整修。新增公交车100部，新增线路5条，设立公交专用道，方便群众出行。畅通农村道路，创建农村公路文明风景线2000公里以上。继续实施饮水安全工程，全面解决农村剩余143个村街、16.1万居民以及61所学校、3.3万师生的饮水安全问题。繁荣农村商贸，促进鲜活农产品流通升级，便民农家店覆盖三分之一村街。完成23个空白乡镇邮政局所补建任务。市新殡仪馆开工建设。

扶贫帮困提升工程。积极帮扶城乡困难群众、特困家庭，不让一名群众因贫无助，不让一个学生因贫失学。“阳光低保”实现各县（市区）和所有低保户100%双覆盖，探索建立计生特殊家庭养老长效救助机制。建立困难残疾人生活和重度残疾人护理补贴制度，为全部符合条件的聋儿提供人造耳蜗手术补贴，建设3-5个残疾人康复示范基地，完成4000名残疾人培训，扶持6000名农村残疾人稳定就业。

文体惠民提升工程。以市县乡村“两馆一站一室”和乡村两级文化队伍建设为重点，确保国家公共文化服务体系示范区创建成功。组织好优秀美术摄影巡展、“幸福廊坊”文化艺术节、特色文化博览会、合唱艺术节等文化活动，丰富群众文化生活。完善健身设施，市区18个游园新增健身设施77套，全市420个村新增健身设施3360件，服务群众60万人。

公共安全提升工程。强力开展食药安全专项整治行动，对重点企业实现从生产到销售全过程监管。强力开展安全生产隐患治理集中行动，深化重点领域专项整治，坚决遏制重特大事故发生。强力开展打黑除恶扫痞灭霸集中行动，净化社会环境。做好信访工作，畅通和规范群众诉求表达、利益协调、权益保障渠道，用法治思维和法治方式化解社会矛盾。

加强国防后备力量建设，大力开展双拥共建，支持工会、共青团、妇联等人民团体发挥桥梁纽带作用，做好新闻广电出版、民族宗教、外事侨务、人民防空、气象地震、防灾减灾、史志档案、援疆等工作。高质量编制“十三五”规划，促进各项事业协调发展。

四、全面加强法治政府建设

全面落实《推进法治廊坊建设的实施意见》，切实推进依法行政，不断提高政府工作法治化水平。

一是切实依法履职。健全政府学习法律制度安排。加快政府职能转变，把工作重心转移到提供公共服务、营造市场环境、维护公平正义上来。严格依照法律程序行使权力，坚决纠正不作为乱作为，坚决克服懒政怠政，坚决惩处失职渎职行为。理顺县级政务服务中心管理体制，完善“三级平台、两个代办”制度，推行政府权力清单、责任清单和负面清单制度，不断提高行政效能和为民服务水平。

二是强化依法决策。完善重大行政决策程序，切实把公众参与、专家论证、风险评估、合法性审查、集体讨论决定作为行政决策法定程序，提高行政决策的规范化、民主化、法治化水平。健全决策合法性审查机制和政府法律顾问制度。实施重大行政行为备案办法，建立重大决策终身责任追究制度和责任倒查机制。加强行政决策落实监督，建立健全定期考核、调度、督导落实机制，确保决策执行到位。

三是严格公正执法。加快建设权责明确、行为规范、监督有效、保障有力的行政执法体制，整合市县两级政府执法队伍，避免多头执法、重复执法。完善执法程序，细化量化行政裁量标准，提高执法效率和规范化水平。全面落实行政执法责任制，强化执法公开，加强执法监督，完善评议考核，不断规范行政许可、行政处罚、行政强制、行政征收等执法行为。

四是加强权力监督。自觉接受人大法律监督、政协民主监督和社会各界监督。加强审计、监察等政府内部权力制约，重点对权力集中的部门和岗位实行分事行权、分岗设权、分级授权和定期轮岗等制度，强化内部流程控制，使权力在科学制衡下有效行使。积极推进政务公开，让权力在阳光下运行。建立健全行政纠错问责机制，强化政府内部层级监督和行政应诉工作，完善问责方式和程序，加强对违法违纪行为的惩处力度。

五是坚决反腐倡廉。巩固和深化党的群众路线教育实践活动成果，落实反“四风”长效机制，严格执行“八项规定”和“约法三章”。健全厉行节约反对浪费制度体系，坚决控制一般性支出，确保“三公”经费只减不增。完成机关公务用车制度改革。全面推进惩治和预防腐败体系建设，严格落实党风廉政建设“一岗双责”。严厉查处土地、建设、交通和专项资金使用等重点领域的腐败行为，对腐败分子和损害群众利益行为绝不心慈手软、坚决依法严惩，努力做到干部清正、政府清廉、政治清明。

各位代表，廊坊的发展已经站在新的历史起点上，今天的廊坊正处在抢抓大机遇、集中发大力、体现大作为的重要阶段，实现跨越赶超是我们肩负的历史责任。让我们紧密团结在以习近平同志为总书记的党中央周围，在省委、省政府和市委的坚强领导下，团结拼搏，奋发进取，大干实事，实干大事，全力夺取2015年各项工作的新胜利，为廊坊绿色发展、高端发展、率先发展、和谐发展作出新的更大的贡献！
